# Algorithm and software to automatically identify latency and amplitude features of local field potentials recorded in electrophysiological investigation

**DOI:** 10.1186/s13029-017-0062-5

**Published:** 2017-02-07

**Authors:** Maria Rubega, Claudia Cecchetto, Stefano Vassanelli, Giovanni Sparacino

**Affiliations:** 1 Department of Information Engineering, University of Padova, Padova, 35131 Italy; 2 Department of Biomedical Sciences, University of Padova, Padova, 35131 Italy

**Keywords:** LFP, Automated analysis, Rat barrel cortex, Whisker stimulation, Phillips-Tikhonov regularization, Neuroscience

## Abstract

**Background:**

Local field potentials (LFPs) evoked by sensory stimulation are particularly useful in electrophysiological research. For instance, spike timing and current transmembrane current flow estimated from LFPs recorded in the barrel cortex in rats and mice are exploited to investigate how the brain represents sensory stimuli. Recent improvements in microelectrodes technology enable neuroscientists to acquire a great amount of LFPs during the same experimental session, calling for algorithms for their quantitative automatic analysis. Several computer tools were proposed for LFP analysis, but many of them incorporate algorithms that are not open to inspection or modification/personalization. We present a MATLAB software to automatically detect some important LFP features (latency, amplitude, time-derivative value in the inflection-point) for a quantitative analysis. The software features can be customized by the user according to his/her personal research needs. The incorporated algorithm is based on Phillips-Tikhonov regularization to deal with noise amplification due to ill-conditioning. In particular, its accuracy in the estimation of the features of interest is assessed in a Monte Carlo simulation mimicking the acquisition of LFPs in different SNR (signal-to-noise-ratio) conditions. Then, the algorithm is tested by analyzing a real set of 2500 LFPs recorded in rat after whisker stimulation at different depths in the primary somatosensory (S1) cortex, i.e., the region involved in the cortical representation of touch in mammals.

**Results:**

Automatic identification of LFP features by the presented software is easy and fast. As far as accuracy is concerned, error indices from simulated data suggest that the algorithm provides reliable estimates . Indeed, results obtained from LFPs recorded in rat after whisker stimulation are in line with the known sequential activation of the microcircuits of the S1 cortex.

**Conclusion:**

A MATLAB software implementing an algorithm to automatically detect the main LFPs features was presented. Simulated and real case studies showed that the employed algorithm is accurate and robust against measurement noise. The available code can be used as it is, but the reported description of the algorithms allows users to easily modify the code to cope with specific requirements.

**Electronic supplementary material:**

The online version of this article (doi:10.1186/s13029-017-0062-5) contains supplementary material, which is available to authorized users.

## Background

Local field potentials (LFPs) reflect the synchronized population activity of several neurons recorded by small-size electrodes in the brain. Any kind of transmembrane current in brain cells contributes to the extracellular fields known as LFPs. Thus, the amplitude and frequency of LFPs depend on the proportional contribution of the multiple sources and various properties of the brain tissue and its cells [1]. A quantitative analysis of LFPs is important in many neuroscience investigations. For instance, an interesting case study concerns how rats and other nocturnal animals process information about the spatial coordinates of objects and their identity by seeking out and palpating objects with their whiskers [2], a paradigm believed to be useful for studying how the brain represents sensory stimuli, also in human beings [3]. In particular, experiments in awake rats have demonstrated that the barrel cortex processes the whisking signal [4], while in anesthetized animals millisecond-scale S1 firing patterns encode whisker stimuli [5]. In such studies, LFP signals are usually recorded from a barrel column of the rat S1 cortex using neural probes with multiple recording sites at different depths [6].

**Fig. 1:**
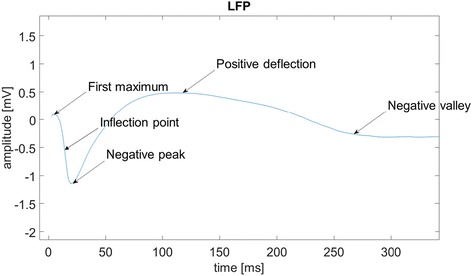
An ideal (noise free) LFP profile after stimulation. *Arrows* indicate some LFP features of interest

A representative example of LFP recording is displayed in Fig. 1, where t = 0 is the time of stimulus occurrence, followed by a first maximum (approximately at t = 8 ms in Fig. 1), an inflection (t = 10 ms), an evident negative peak (t = 20 ms), a slow positive deflection (t in [60, 200] ms) and a slow long negative valley (t in [200, 500] ms) that goes ahead of a gradual restore of the baseline (t > 500 ms). To provide information about the propagation time required for the stimulus to reach the layer of LFP recording, the investigator is interested in the latency (and the amplitude) of the first maximum, which can be identified as the stimulus onset. Moreover, particular attention is paid to latency and amplitude of the negative peak, which is associated with layer activation [7]. The determination of the inflection point between the first maximum and the negative peak is also useful to measure the decreasing rate of the signal between the onset and the main negative peak.

Visual identification and analysis of LFP features are obviously time consuming and exposed to the risk of subjectivity. Nowadays, a large number of LFPs (e.g. 500) can be collected within a single experimental session thanks to improvements in microelectrodes technology, making the development of algorithms for LFP quantitative automatic analysis even more urgent. Indeed, a number of algorithms and software tools for LFP analysis is available. For instance, in [8, 9], the software returns the location of the peaks at which data exceed an imposed threshold. In [10], spikes are first detected by setting an amplitude threshold, then are clustered by exploiting wavelet coefficients [12] or PCA (principal components analysis). In another software program [11], a graphical application for manual spike sorting is provided.

A limitation of many software tools for LFP analysis is that algorithmic details are not fully described and/or codes are not open to customization according to specific investigational needs. In the present paper, we illustrate a software to automatically detect some features of LFP waveforms pointed out in Fig. 1 named first maximum, inflection point and negative peak. The time-derivatives used by the core algorithm to determine the features are calculated by the Phillips-Tikhonov regularization method which mitigates measurement noise amplification due to ill-conditioning [13]. Several parameters of the MATLAB software can be customized by the user for personal research needs. The accuracy of the algorithm in correctly estimating the mentioned LFP features is first assessed in a Monte Carlo simulation mimicking the acquisition of LFP in different conditions of SNR. Then, the algorithm is tested by analyzing a set of real LFPs recorded in the S1 cortex of a rat at five different depths after whisker mechanical stimulation.

## Methods

### Brief description of the algorithm

With reference to the ideal case (noise free) of Fig. 1, the main goals are to detect: latency and amplitude of the first maximum (identified as the stimulus onset in this work), latency and amplitude of the negative peak, and first derivative value of the inflection point. In real world, recorded LFP are affected by measurement error and the automatic determination of their minima, maxima, and inflection points is complicated by the presence of noise which is amplified when time-derivatives are computed because of ill-conditioning. This makes the straightforward use of techniques employed in other contexts difficult, e.g. for the automatic analysis of latencies of event-related potentials [14, 15].

In order to deal with ill-conditioning, estimation of first and second time derivatives, needed to identify minima, maxima and inflection points, was achieved by the well-known Phillips-Tikhonov regularization approach. Detailed description of this method can be found in several papers, e.g., [16], thus only a brief summary of the main steps is reported. The vector **y** contains the N equally spaced samples of the recorded noisy LFP. The following relation holds

1 $$ \mathbf{y}=\mathbf{G}\mathbf{u}+\mathbf{v} $$

where **u** is an N-dimensional vector containing the levels on the sampling grid of the LFP time-derivative to estimate. **v** is the N-dimensional measurement noise vector. **G** is a NxN lower triangular Toeplitz matrix whose first column is [1, 1, ⋯,1] ^T^ if **u** stands for the first time-derivative, and [1, 2, 3, ⋯, N] ^T^ if **u** stands for the second time-derivative. Vector **v** is assumed to be uncorrelated, with zero mean, and standard deviation σ, whose value can be numerically estimated from samples of the baseline signal (once and for all sweeps). According to Phillips-Tikhonov regularization, an estimate of **u** can be obtained from **y** as

2 $$ \hat{\mathbf{u}}={\left({\mathbf{G}}^T\mathbf{G}+\upgamma {\mathbf{F}}^{\mathbf{T}}\mathbf{F}\right)}^{-1}{\mathbf{G}}^{\mathrm{T}}\mathbf{y} $$

where **F** is a N-dimension lower triangular Toeplitz matrix having [1, −2, 1, 0, …, 0] ^T^ as first column and γ is an unknown positive parameter whose value is found by trials until the so-called “discrepancy” regularization criterion [17] is satisfied.

To speed up the determination of γ, the numerical procedure described in detail in [16] is used. Briefly:

1. The singular value decomposition of matrix **H** = **GF** ^−1^ is performed to obtain N-size unitary matrices **U** and **V** and matrix **D** = diag ([d_1_, d_2_, …, d_N_]^T^)
  3 $$ {\mathbf{U}}^{\mathrm{T}}\mathbf{H}\mathbf{V}=\mathbf{D} $$
  This operation, which is performed only once, has numerical complexity O (N^3^).
2. γ is tuned until the condition of the discrepancy regularization criterion is met, i.e., until the following equation
  4 $$ \mathrm{WRSS}={\displaystyle {\sum}_{i=1}^N{\left(\frac{\gamma {\upxi}_{\mathrm{i}}}{{d_i}^2+\gamma}\right)}^2} $$
  is (approximately) matched, where **ξ** = **U** ^T^ **y** and WRSS stands for weighted residual sum of squares. As explained in [16], for each trial value of γ, the numerical complexity of this stage is only O (N).
3. As γ is determined, the vector of the unknown time-derivative samples **û** is computed as
  5 $$ \hat{\mathbf{u}}={\mathbf{F}}^{-1}\mathbf{V}\mathbf{n} $$
  where **n** = [n_1_, n_2_, …, n_i_, …, n_N_] with ${\mathrm{n}}_{\mathrm{i}}=\frac{{\mathrm{d}}_{\mathrm{i}}\;\upxi \mathrm{i}}{{{\mathrm{d}}_{\mathrm{i}}}^2+\upgamma}$. This stage has O (N^3^) complexity.
  As by-product of the procedure, a regularized version of the LFP is obtained by multiplying **û** by matrix **G**. The difference between **y** and **Gû** over σ is the vector of the normalized residuals.

After the regularization step, the unknown first maximum and negative peak of the LFP are determined by finding where the estimated first time-derivative crosses the zero line (change in the sign is also required). To make their estimation more robust avoiding the possibility to estimate a local minimum instead of the global one, a minimal distance (determined by the experimenter) between them is imposed. After estimating the first maximum and the negative peak, the onset is computed between them (the relative position of the onset is also forced by the user). Finally, the inflection point is estimated by finding where the second time-derivative goes to zero in the interval between the previously determined first maximum and the negative peak (presence of change in its sign in the neighborhood interval is also verified).

### MATLAB implementation

The software was developed in MATLAB R2014b. The source code files and a limited set of data to test the software are provided as additional files of the presented paper. To facilitate people who do not want to handle MATLAB code, a GUI is provided to guide the user step-by-step in inserting the information requested during the running of the software. Briefly, the user has to specify:

- ■ The path of the folder containing the data.
- ■ The name of the .mat file containing the data.
- ■ The name of the experiment (used to name the .mat file and .xls file that will contain the extracted features).
- ■ The depth of recording in order to name the .xls sheet (thus the .xls file will contain a sheet for each depth of recording referred to the same experiment).
- ■ The sampling frequency of the data.
- ■ The starting and the finishing time of the window of analysis.
- ■ The factor n to possibly decrease the sampling rate (if the sampling frequency of the acquired data is unnecessarily high for the purpose).
- ■ The position of the onset between the first maximum and the main negative peak, expressed in the range [0 1]. A latency parameter is computed by subtracting the stimulus onset to the negative peak time.
- ■ The minimum distance hypothesized by the experimenter from the onset and the main negative peak.

The .mat file containing the data has to be organized as follows: a matrix, in which each column stands for a single recording, and a time vector in ms (Additional file 1). If the data are not in the .mat format, a script to convert .txt file to .mat file is provided. The .txt file has to be structured in columns, in which the first one contains the time vector and the others the amplitudes of the recordings (Additional file 2; Additional file 3). Eventually, the software displays the results, and produces a .mat file and a .xls file containing the features extracted. This approach lets the user import the results into MATLAB or in Microsoft Excel for a further off-line processing.

## Results

### Software use in a representative case study

**Fig. 2:**
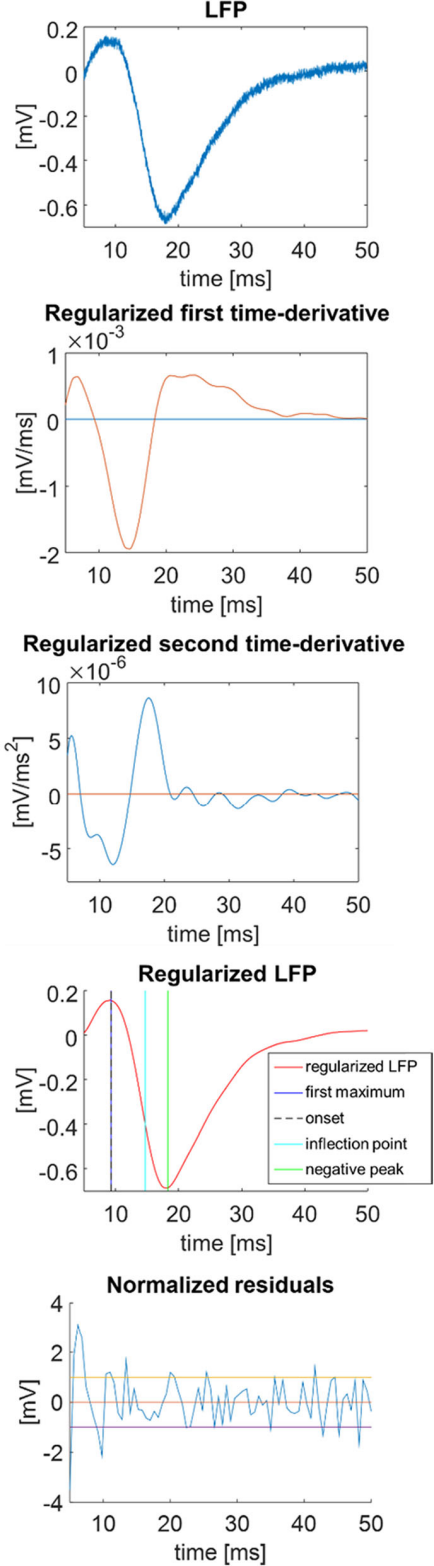
Example of algorithm outcome. Starting from top, a representative raw LFP, the regularized first time-derivative, the regularized second time-derivative, the regularized LFP and the estimated points, the normalized residuals

**Fig. 3:**
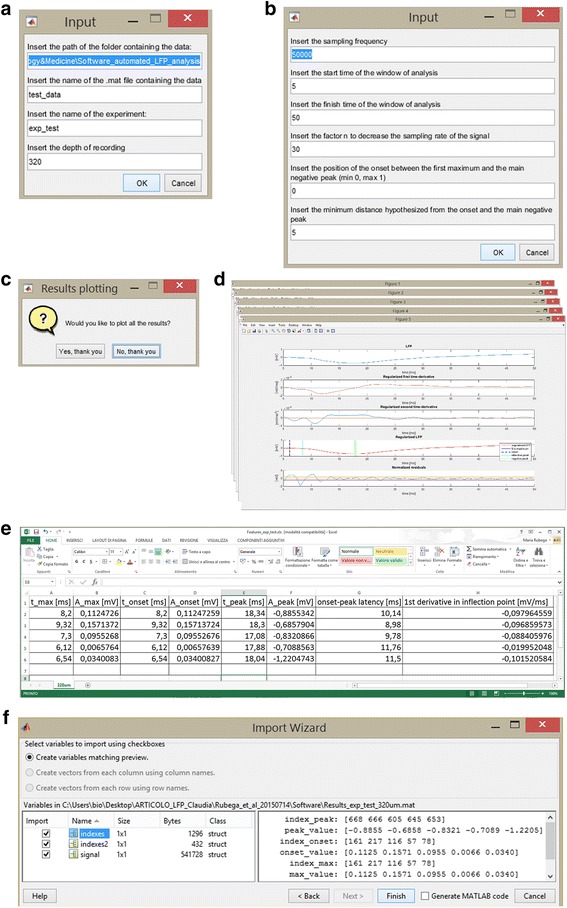
The software GUI. In panels **a** and **b**, GUI that acquires the experimental parameters from the user. In panel **c**, plot of the results reported also in Fig. 2. In panel **d**, user's choice to plot all the results. In panel **e**, example of the .xls file generated as output (in each column, there are respectively latency and amplitude of the first maximum (t_max_, Amax), of the onset (t_onset_, A_onset_), and of the negative peak (t_peak_, A_peak_) and, value of the first time-derivative of the inflection point). In panel **f**, example of the respective .mat file (the struct *indexes* contains the estimates of the first maximum, the inflection point and, the negative peak, the struct *indexes2* the estimates of the inflection point and of its first-time derivative value and, finally, the struct *signal* contains the smooth LFP and its time-derivatives)

The main aim of this section is to detect the negative peak associated with layer activation visible in Fig. 1. The window of analysis is limited from 5 ms to 50 ms after the whisker stimulation (the first 5 ms are ignored because of the usual presence of a spike artifact caused by the electro-mechanical stimulation). Fig. 2, upper panel, shows a representative raw LFP signal measured at 720 μm of depth after whisker stimulation. In contrast to the ideal case depicted in Fig. 1, the presence of noise is evident. Figure 3 shows how the provided GUI acquires the experimental parameters from the user (panels a and b) and returns the results (panels d, e and f). In our experiment, as visible in Fig. 3 panel b, the sampling frequency was equal to 50 kHz, thus it was down-sampled with a factor of 30 and, the first maximum was individualized as the onset. All the parameters of our experimental setup can be easily modified by non–programming users when the software starts. For sake of graph readability, the results of panel d are also reported, with a different layout, in Fig. 2. Starting from the top, the first panel reports the raw LFP sweep. The second and the third panels show, respectively, the regularized estimates of the first and second time-derivatives employed in the computation of the LFP features. The fourth panel displays the regularized version of the LFP with the identified features (first maximum, inflection point and negative peak, with values that can be exported as a table in an .xls file and in a .mat file, see Fig. 3 panel e and f). Eventually, the fifth panel reports the weighted residuals (in this case σ was 0.005) which appear approximately uncorrelated and mostly lying in the ±1 interval.

To allow more flexibility than that allowed by the GUI, the software was intentionally organized in nested functions that investigators with basic knowledge of MATLAB can easily customize. Indeed, the main script (Additional file 4) calls two different functions (Additional file 5; Additional file 6), where the signal smoothing and the computation of its first and second time-derivatives are performed following the stages described in the Methods section (see also Additional file 7; Additional file 8) . Thus, it is simple to do some little tweaks, e.g., changing the value of σ, and/or some bigger tweaks, e.g., modifying the criterion for the estimation of γ.

### Reliability of the algorithm and robustness against SNR (simulated data)

**Fig. 4:**
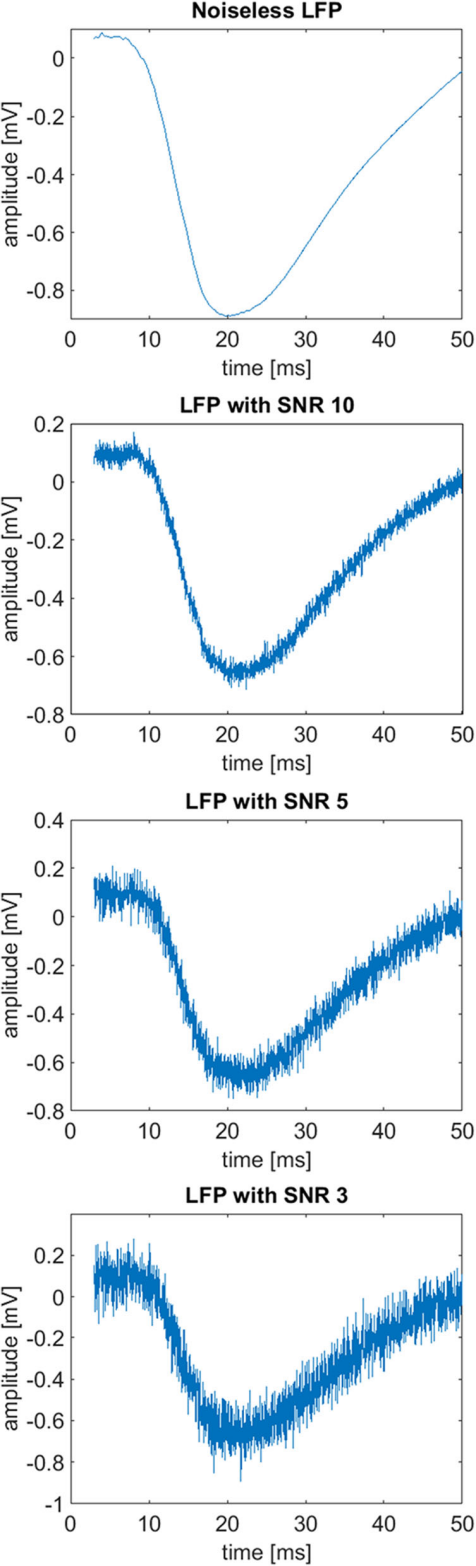
Simulated data. Starting from top, noiseless LFP and LFP with increasing level of noise (SNR equal to 10, 5 and 3)

To give an idea of the reliability of the algorithm in the considered paradigm, i.e., LFP measured in rat after whisker stimulation, the performance of the method was assessed on simulated data. To better reproduce the variety of situations which can be encountered in real data, seven possible reference LFP profiles were considered as ground truth. Each of these reference profiles was built (details not reported) from real LFP recordings performed in the barrel column of a rat S1 cortex at different depths after whisker stimulation. In particular, each reference profile stands for the typical noiseless template of recordings from 320 to 920 μm, with step of 100 μm of depth. For instance, Fig. 3 (top panel) displays noiseless LFP. Noiseless data were considered as ideal case (SNR ⟶ Inf). Then the deterioration of the performance with progressively increasing level of noise (SNR equal to 10, 5 and 3) was observed. For instance, in Fig. 4, middle and bottom panels, we report three examples of LFP signal with SNR equal to 10, 5 and 3, respectively, where SNR is determined as

6 $$ \mathrm{S}\mathrm{N}\mathrm{R}=\frac{\mathrm{s}{{\mathrm{d}}^2}_{\mathrm{LFP}}}{\mathrm{s}{{\mathrm{d}}^2}_{\mathrm{noise}}} $$

where *sd* ^*2*^_*LFP*_ stands for the variance of the LFP signal and *sd* ^*2*^_*noise*_ for the variance of the noise. For each reference LFP, for the chosen SNR level, 100 different noisy sweeps, i.e., y of Eq. (1), were created by adding 100 different artificially generated noise sequences v. From each sweep, the following LFP features were extracted by means of the algorithm described in Methods section: latency and relative amplitude of the first maximum (t_max_, A_max_) and of the negative peak (t_peak_, A_peak_), and value of the first time-derivative of the inflection point. To evaluate the performance of the method, these values were compared with those estimated from the noiseless LFP. Eventually, the six error indices were calculated as the average over all the sweeps of the difference. In particular, the difference referred to the amplitude of the first maximum and of the negative peak and to the first time-derivative in the inflection point was normalized to the respective true value in order to obtain an error index in the range [0 1].

**Fig. 5:**
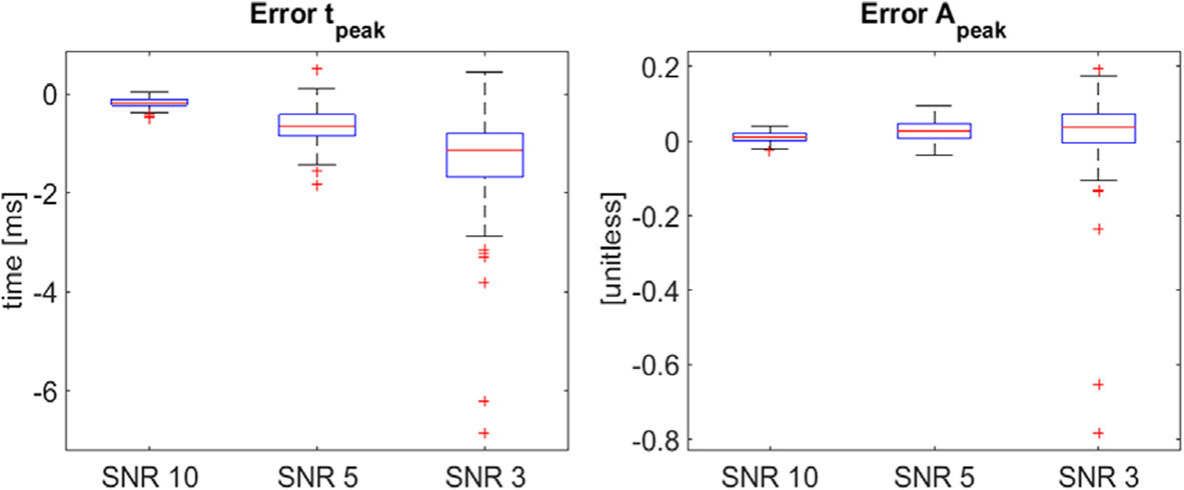
Distribution of errors in estimating time and amplitude of the main negative peak. *Errors* are computed from noisy signals referred to 720 μm of depth for progressively deteriorating SNR. In each *boxplot*, the *central red line* is the median value, the edges of the box are the 75th and 25th percentiles, the whiskers extend to the most extreme data points that are not outliers, with outliers being plotted individually by *red crosses*

The boxplots of Fig. 5 illustrate two examples of the distributions of the difference between the estimated values and the true values of the time and the amplitude of the negative peak in the noisy signals referred to 720 μm of depth with progressive deteriorating SNR (10, 5 and 3). As expected, the error median value (horizontal red line) tends to 0 and the error variability significantly decreases as the SNR increases. In Table 1, the six error indices are reported for the noisy signals referred to 720 μm of depth. In particular, the 2^nd^ column stands for the error in the estimation of the latency of the first maximum (t_max_); the 3^rd^ column to the error referred to the amplitude of the first maximum (A_max_); the 4^th^ and 5^th^ columns to the errors associated to the latency and amplitude of the main negative peak (t_peak_ and A_peak_). Finally, the 6^th^ column of Table 1 is referred to the error in the estimation of the first-time derivative value in the inflection point. In agreement with Fig. 5, estimates deteriorate as the SNR worsen. Similar results (not shown) were obtained for the signals referred to the other depths.

**Table 1:** Error indices in simulated data

	Error indices				
SNR	t_max_ = t_onset_ [ms]	A_max_ = A_onset_ [unitless]	t_peak_ [ms]	A_peak_ [unitless]	1^st^ derivative value in the inflection point [unitless]
10	0.25 (0.12)	−0.01 (0.14)	−0.16 (0.09)	0.01 (0.01)	0.05 (0.02)
5	0.89 (0.96)	−0.01 (0.31)	−0.64 (0.36)	0.03 (0.02)	−0.21 (0.36)
3	2.77 (1.24)	−0.73 (0.99)	−1.39 (1.09)	0.01 (0.03)	−0.06 (0.39)

Error indices calculated as average over all the 100 simulated sweeps (standard deviation is in brackets) for different values of the SNR

Overall results suggest that the method is sufficiently precise for a reliable automated detection of the LFP main features after whisker stimulation. Algorithm numerical efficiency is acceptable, e.g., execution time to process the 100 LFP recordings resulted 714 ms using an Intel Core i7-4790 at 3.6 GHz, with 16 GB of RAM.

### Test on experimental data

**Fig. 6:**
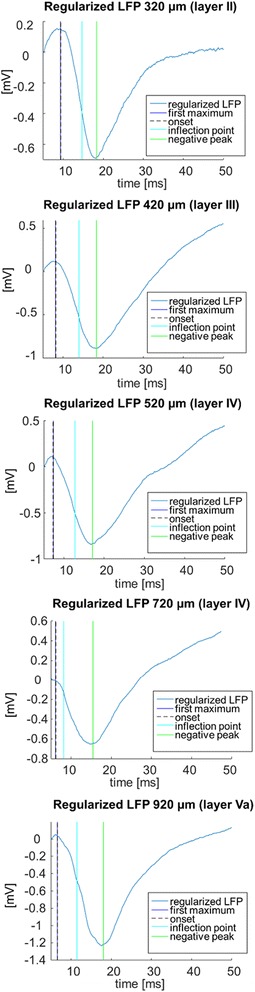
Real LFPs referred to different depths of recording. Starting from top, LFP recorded at 320 μm of depth up to LFP recorded with increasing level of depth (respectively, 420 μm, 520 μm, 620 μm, 720 μm and, 920 μm)

The algorithm was tested on empirical electrophysiological signals recorded from the rat S1 cortex generated by repetitive deflections of rat whiskers to assess the variability in signal shapes and timings. The University of Padova Ethical Committee approved all animal procedures. In these experiments, recordings were performed at 100 μm resolution by means of conventional Ag/AgCl electrodes, starting from 320 μm (Layer II) up to 920 μm (Layer Va) of depth in anesthetized postnatal day 30 (P30) and P50 rats. The cortical area of interest was exposed in correspondence of S1 cortex. At the end of the surgery, single whiskers were deflected repeatedly by means of a piezoeletric bender, triggered by a custom Labview program [18]. At every cortical depth, 500 single sweeps were recorded in response to these mechanical stimulations with a temporal delay between subsequent traces of 2 s to avoid any phenomenon related to adaptation. LFP signals of 500 ms were recorded in response to these mechanical stimulations at 50 kHz of sampling frequency. Then, the software was applied. Figure 6 shows, for each of the considered layers, a representative sweep and the identified features, first maximum, onset, inflection point and negative peak.

A preliminary evaluation was performed by analyzing t_max_, t_peak_ and A_peak_ of the individual LFP responses. The average and the standard deviation on the mean of these parameters computed from 500 single values extracted from the individual LFP responses are reported in Table 2. The relative error on the estimations ranged from 0.3 to 3%, but it was also due to the intrinsic variability of neural signals. Comparing the latencies and the amplitudes of the evoked responses through the different cortical layers, we noticed that the minimum onset and peak latency (t_max_ and t_peak_) and the maximum amplitude of the negative peak (A_peak_) were in correspondence of layer IV. This finding reflects the structure and the inter-layer connectivity of the cortical network [19] and is in line with the known sequential activation of the microcircuits of the barrel [20, 21, 22]. In fact, the neuronal signal coming from the whisker mechanoreceptors travels through the trigeminal ganglion, the brainstem and then conveyes to the thalamus. The neuronal information is mainly transmitted from this structure to layer IV which is therefore the first station of signal processing in the barrel cortex. The neural signal is then transferred to the superficial (layer II and III) and deeper layers (layer V) through complex cortical projections. Thus, longer activation timings, i.e., onset and peak latencies, and smaller responses, i.e., peak amplitudes, were expected for these cortical layers.

**Table 2:** Main features automatically detected in real data

	Main features automatically extracted				
Depth [μm]	t_max_ = t_onset_ [ms]	A_max_ = A_onset_ [mV]	t_peak_ [ms]	A_peak_ [mV]	1^st^ derivative value in the inflection point [mV/ms]
320 (layer II)	10.92 (0.09)	0.086 (0.002)	19.80 (0.11)	0.607 (0.010)	−0.038 (0.001)
420 (layer III)	9.43 (0.04)	0.035 (0.002)	18.26 (0.06)	1.070 (0.015)	−0.064 (0.002)
520 (layer IV)	8.76 (0.11)	0.022 (0.004)	18.75 (0.16)	1.005 (0.019)	−0.064 (0.001)
720 (layer IV)	8.04 (0.04)	−0.008 (0.001)	17.29 (0.05)	1.122 (0.019)	−0.069 (0.002)
920 (layer Va)	8.20 (0.30)	−0.006 (0.005)	18.2 (0.3)	0.764 (0.018)	−0.026 (0.001)

Mean values (and standard deviation in brackets) of the main features of the response automatically extracted through the software from 2500 LFP signals recorded at different recording depths

## Conclusions

In this paper, we presented a MATLAB software, exploiting Phillips-Tikhonov regularization to automatically detect the first maximum, the following negative peak, and the first time-derivative value in the inflection point between them, in LFPs evoked by whisker stimulation in rat barrel cortex. Preliminary experimental tests performed on simulated data with an increasing level of noise proved that the algorithm can be successfully used in automatically estimating the features of interest. Moreover, the method was successfully exploited to analyse a large LFP dataset to evaluate the differences in the response at different recordings depths. The codes were designed to allow customization by the user. Thus, on one hand, the investigator without programming background can customize the analysis by easily setting the main parameters to guide the features estimation and visualize its results, on the other hand, the programming user can modify the code exploiting its nested organization.

Current activity regards the massive analysis of large LFP datasets and their physiological interpretation. Moreover, the algorithm may be extended to estimate other features of interest, such as the duration of the positive deflection and of the negative valley of Fig. 1.

## Additional files

Additional file 1: Which each column stands for a single recording in mV, a time vector (17500x1) in ms, and a structure containing the sampling frequency and the sampling interval. (MAT 94 kb)
Additional file 2: Txt file containing in the first column the time vector in ms, in the second and third ones the recordings in mV. (TXT 781 kb)
Additional file 3: A script that convert .txt file (containing data) into .mat file. The .txt file must be organized in columns, in which the first one contains the time vector and the others the amplitudes of the recordings. The .mat file will contain a matrix RAT (number of samples x number of sweeps), a vector new_time (number of samples × 1) and a struct parameters (parameters.dT: sampling interval; parameters. Fs: sampling frequency; parameters. Ns: number of samples for each sweep). (M 1 kb)
Additional file 4: The main script that visualizes the smoothing of the signal, its first/s time-derivative, and the other features of interest (maximum, onset, inflection point). All results are saved in an .xls file (each sheet contains the results relative to a particular depth) and in a .mat file. (M 6 kb)
Additional file 5: A function that is called by main_script.m to compute the smoothing of the signal and its first-time derivative. Also the functions that guarantee the correct running of main_script.m. To test the algorithm, invoking only main_script.m is necessary (all the other functions must be contained in the same folder). (M 1 kb)
Additional file 6: A function that is called by main_script.m to compute the signal second-time derivative. Also the functions that guarantee the correct running of main_script.m. To test the algorithm, invoking only main_script.m is necessary (all the other functions must be contained in the same folder). (M 1 kb)
Additional file 7: A function that is called by main_script.m to compute the onset and the maximum latencies and amplitudes from the signal time-derivative. Also the functions that guarantee the correct running of main_script.m. To test the algorithm, invoking only main_script.m is necessary (all the other functions must be contained in the same folder). (M 1 kb)
Additional file 8: A function that is called by find_negativepeak_onset_max.m to compute the peak latency and amplitude. Also the functions that guarantee the correct running of main_script.m. To test the algorithm, invoking only main_script.m is necessary (all the other functions must be contained in the same folder). (M 874 bytes)

## Abbreviations

A_max_: Amplitude of the first maximum
A_onset_: Amplitude of the onset
A_peak_: Amplitude of the negative peak
Inf: Infinity
LFPs: Local field potentials
P: Postnatal day
PCA: Principal Components Analysis
S1: Primary somatosensory cortex
sd^2^: Variance
SNR: Signal-to-noise ratio
t_max_: Latency of the first maximum
t_onset_: Latency of the onset
t_peak_: Latency of the negative peak
